# Arginase Activities and Global Arginine Bioavailability in Wild-Type and ApoE-Deficient Mice: Responses to High Fat and High Cholesterol Diets

**DOI:** 10.1371/journal.pone.0015253

**Published:** 2010-12-06

**Authors:** Aaron Erdely, Diane Kepka-Lenhart, Rebecca Salmen-Muniz, Rebecca Chapman, Tracy Hulderman, Michael Kashon, Petia P. Simeonova, Sidney M. Morris

**Affiliations:** 1 Toxicology and Molecular Biology Branch, National Institute for Occupational Safety and Health, Morgantown, West Virginia, United States of America; 2 Laboratory for Occupational Cardiovascular Toxicology, National Institute for Occupational Safety and Health, Morgantown, West Virginia, United States of America; 3 Biostatistics and Epidemiology Branch, Health Effects Laboratory Division, National Institute for Occupational Safety and Health, Morgantown, West Virginia, United States of America; 4 Department of Microbiology and Molecular Genetics, University of Pittsburgh School of Medicine, Pittsburgh, Pennsylvania, United States of America; Biological Research Center of the Hungarian Academy of Sciences, Hungary

## Abstract

Increased catabolism of arginine by arginase is increasingly viewed as an important pathophysiological factor in cardiovascular disease, including atherosclerosis induced by high cholesterol diets. Whereas previous studies have focused primarily on effects of high cholesterol diets on arginase expression and arginine metabolism in specific blood vessels, there is no information regarding the impact of lipid diets on arginase activity or arginine bioavailability at a systemic level. We, therefore, evaluated the effects of high fat (HF) and high fat-high cholesterol (HC) diets on arginase activity in plasma and tissues and on global arginine bioavailability (defined as the ratio of plasma arginine to ornithine + citrulline) in apoE^−/−^ and wild-type C57BL/6J mice. HC and HF diets led to reduced global arginine bioavailability in both strains. The HC diet resulted in significantly elevated plasma arginase in both strains, but the HF diet increased plasma arginase only in apoE^−/−^ mice. Elevated plasma arginase activity correlated closely with increased alanine aminotransferase levels, indicating that liver damage was primarily responsible for elevated plasma arginase. The HC diet, which promotes atherogenesis, also resulted in increased arginase activity and expression of the type II isozyme of arginase in multiple tissues of apoE^−/−^ mice only. These results raise the possibility that systemic changes in arginase activity and global arginine bioavailability may be contributing factors in the initiation and/or progression of cardiovascular disease.

## Introduction

Arginine metabolism plays important roles in vascular function in health and disease. The key enzymes involved in arginine metabolism in the vasculature are the nitric oxide synthases and the arginases, both of which use arginine as a substrate [1], and dysregulated activity or expression of these enzymes has been linked to multiple types of endothelial dysfunction and cardiovascular disease [2]–[5]. Because diet is one of the major modifiable risk factors for cardiovascular disease, there is considerable interest in characterizing the impact of high-risk diets on changes in arginine metabolism that are associated with cardiovascular disease.

One example of cardiovascular disease that involves dysregulated arginine metabolism and also is strongly influenced by diet is atherosclerosis. Stages of atherogenesis, more specifically endothelial dysfunction, have been strongly related to dysregulated nitric oxide (NO) synthesis [6]. Nitric oxide production by the enzyme nitric oxide synthase in the vascular endothelium requires the substrate L-arginine. Thus, L- arginine supports antiatherogenic effects mostly mediated by nitric oxide. However, L- arginine is also catabolized by arginase to ornithine and urea. Arginase, induced in response to inflammatory stimuli in a variety of cell types [7]–[9], consists of two isozymes (types I and II), and there is increasing evidence that arginase is a biologically relevant modulator of vascular structure and function. In models of endothelial dysfunction as a consequence of aging, ischemia-reperfusion or hypertension, vascular arginase has been shown to be elevated [10]–[16]. Both arginase inhibition and arginine supplementation restore vascular function and nitric oxide production, indicating a role of substrate limitation by arginase for endothelial nitric oxide synthase [10]–[13], [15], [16]. Furthermore, *in vitro* studies have shown that arginine metabolism via arginase in vascular smooth muscle cells increased downstream polyamine and proline synthesis, resulting in increased proliferation and collagen synthesis [17]–[19]. Therefore, increased arginase can contribute to vascular disease by both reducing available arginine for NO synthesis and enhancing vascular remodeling. The roles and regulation of arginase activity and systemic L-arginine metabolism during initiation and progression of cardiovascular diseases, such as atherosclerosis, are not well understood. More specifically, the effects of diets with varying lipid and cholesterol content on systemic arginase activity and global arginine bioavailability have not been evaluated.

We hypothesized that high fat diets—with and without high cholesterol—would cause systemic disruption of arginine metabolism through increased arginase activity. We also hypothesized that a high fat-high cholesterol diet that leads to atherosclerosis in an animal model would result in changes in arginine metabolism or arginase expression beyond that resulting from a high fat diet alone. To test these hypotheses, apoE^−/−^ mice, genetically prone to develop atherosclerosis [20], and C57BL/6 mice, the wild type controls, were fed a standard chow diet, a high fat-high cholesterol (1.25%) diet containing cholate (HC), known as a highly atherogenic diet [21], and a high fat (HF) diet with moderate cholesterol (0.2%), associated with obesity rather than with atherosclerosis [22]. We evaluated plasma arginase activity, plasma lipids and amino acids, plasma levels of liver enzymes, and arginine/(ornithine + citrulline) ratio, an indicator of global arginine bioavailability [23], [24]. The activities and expression of the arginases also were evaluated in multiple tissues.

## Results

### Effects of diets on plasma enzyme activities and plasma lipids

**Figure 1:**
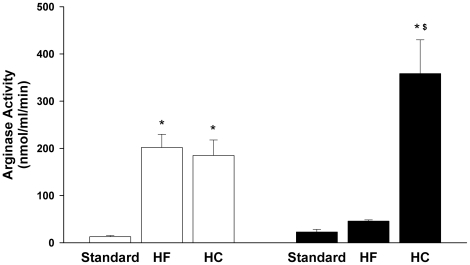
Effect of HF and HC diets on plasma arginase activity (nmol/ml/min). Values are means ± SE for n = 10-15 for standard and HC diet and n = 6 for HF diet. Key to symbols: apoE^−/−^ (open bars); C57BL/6J (black bars); *p<0.05 vs standard diet; ^$^p<0.05 vs strain-specific HF diet.

Most studies of the effects of diets in apoE^−/−^ mice have focused tightly on development and progression of atherosclerotic plaques. However, the HC diet used here and in many other studies also induces inflammatory responses in the liver [25]–[28], raising the possibility that liver damage might result in elevated plasma levels of arginase I and thus reduce circulating arginine levels. Thus, plasma arginase activities were determined for three independent experiments in which C57BL6 and apoE^−/−^ mice were fed standard, HF or HC diets for 2 months as described in Methods. Because the HC diet differed from the standard chow diet with regard to both fat and cholesterol content, the HF diet was included to differentiate any effects of increased fat content alone. Whereas plasma arginase activities did not differ significantly for C57BL6 and apoE^−/−^ mice on the standard chow diet, there were strain-specific differences in responses to the HF and HC diets (Figure 1). Plasma arginase activities were increased equally in apoE^−/−^ mice on HF and HC diets. Although there was a trend toward increased plasma arginase activity in C57BL6 mice on the HF diet, it was not statistically significant. However, there was a dramatic increase in plasma arginase activity in C57BL6 mice fed the HC diet, to a level nearly twice as high as the elevated plasma arginase activity in apoE^−/−^ mice fed the HF or HC diet (Figure 1).

**Figure 2:**
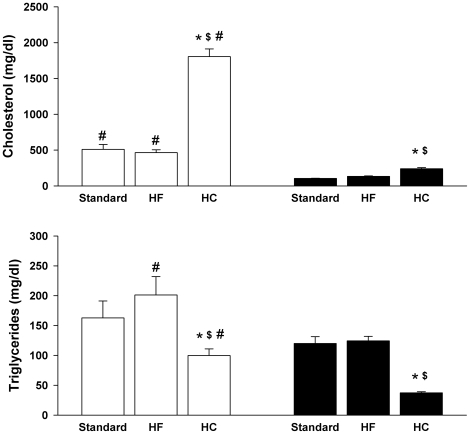
Effect of HF and HC diets on plasma triglycerides and cholesterol. Values are means ± SE for n = 5 mice in each group. Key to symbols: apoE^−/−^ (open bars); C57BL/6J (black bars); *p<0.05 vs strain specific standard diet; ^$^p<0.05 vs strain-specific HF diet; ^#^p<0.05 for apoE^−/−^ vs C57BL/6J fed same diet.

**Figure 3:**
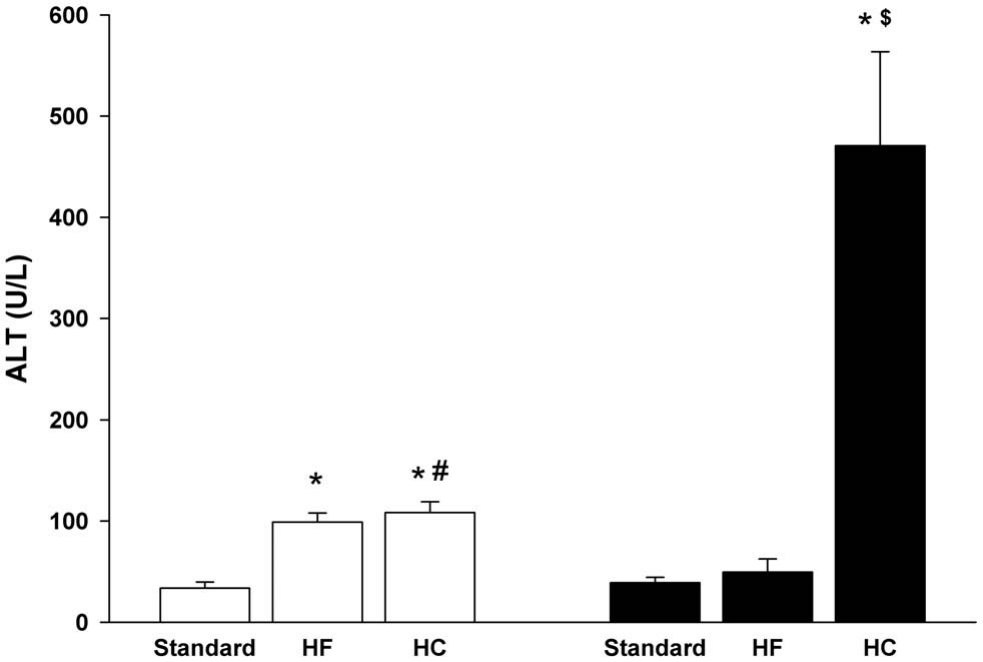
Effect of HF and HC diets on plasma levels of ALT. Values are means ± SE for n = 5 mice in each group. Symbols are defined in Figure 2 legend.

**Figure 4:**
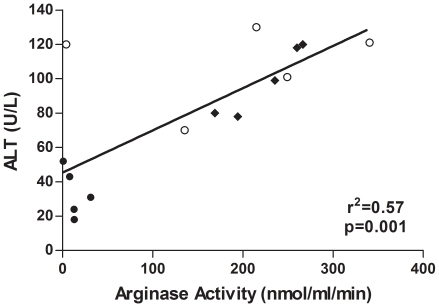
Correlation between activities of ALT and arginase in plasma of apoE^−/−^ mice on different diets. Regression analysis of ALT and arginase activities for the different diets (n = 5 in each group) is indicated by the solid line. Key to symbols: solid circles, standard diet; solid diamonds, HF diet; open circles, HC diet.

As significant diet- and strain-specific differences in plasma arginase activities were found, more detailed biochemical and molecular studies were performed for a representative experiment in which samples of multiple tissues also had been collected. All subsequent data are from that experiment. On all diets, the apoE^−/−^ mice had significantly higher plasma cholesterol levels as compared to C57BL/6J. Whereas the HF diet had no effect on plasma cholesterol, the HC diet induced marked increases in both strains (Figure 2). Plasma triglycerides were significantly reduced by the HC diet in both strains but were not affected by the HF diet in either strain. No changes in blood urea nitrogen were observed in any of the groups, indicating no loss of renal function (data not shown). However, plasma alanine aminotransferase (ALT) levels were significantly increased by the HC diet in both strains but by the HF diet only in the apoE^−/−^ mice (Figure 3). Elevated plasma ALT levels have been shown previously for C57BL/6J mice fed the HC diet [25], [26]. The elevated ALT levels are indicative of liver damage, consistent with previous studies showing liver inflammation and injury associated with high cholesterol-high fat diets in mice [25]–[30]. Notably, the profile of plasma ALT levels closely parallels the profile of plasma arginase activities in Figure 1. For example, plasma ALT and arginase activities are significantly correlated for apoE^−/−^ mice on the three diets (Figure 4), even though apoE^−/−^ mice on the HC diet differ significantly from apoE^−/−^ mice on the standard and HF diets with regard to arginase expression in nonhepatic tissues (discussed below). Because the liver is the greatest source of arginase in the body, the association between the increases in plasma arginase activities and ALT levels indicates that liver damage represents the principal source of increased arginase activity.

### Effects of diets on arginine bioavailability

Plasma levels of arginine, ornithine and citrulline were not significantly affected by any diet in C57BL/6J mice (Table 1). In the apoE^−/−^ mice, however, a reduction in circulating arginine levels was found only with the HC diet. There was also a trend for reduced plasma arginine in apoE^−/−^ mice on the HF diet, but this did not reach statistical significance (p = 0.06; Table 1). Plasma ornithine was significantly increased only in the apoE^−/−^ mice on the HF diet. There were no diet- or strain-specific differences in plasma citrulline levels, suggesting no difference in activity of the intestinal-renal axis of citrulline/arginine metabolism [1]. Overall, there was no consistent pattern of dietary lipid effect on plasma levels of these individual amino acids.

**Table 1:** Effect of diet on plasma amino acid levels (µM) in C57BL/6J and apoE^−/−^ mice.

Mouse strain and Diet	Arginine	Ornithine	Citrulline
C57BL6 Standard diet	115±38	71±26	76±22
C57BL6 HF Diet	91±4	66±7	69±3
C57BL6 HC Diet	85±19	85±11	92±8
			
apoE^−/−^ Standard diet	100±6	61±4	65±2
apoE^−/−^ HF Diet	82±7	85±7*	76±2
apoE^−/−^ HC Diet	73±10*	62±10	76±9

n = 4 for HF groups and n = 3 for all other groups. Values are means ± SE. *p<0.05 vs strain-specific standard diet.

**Figure 5:**
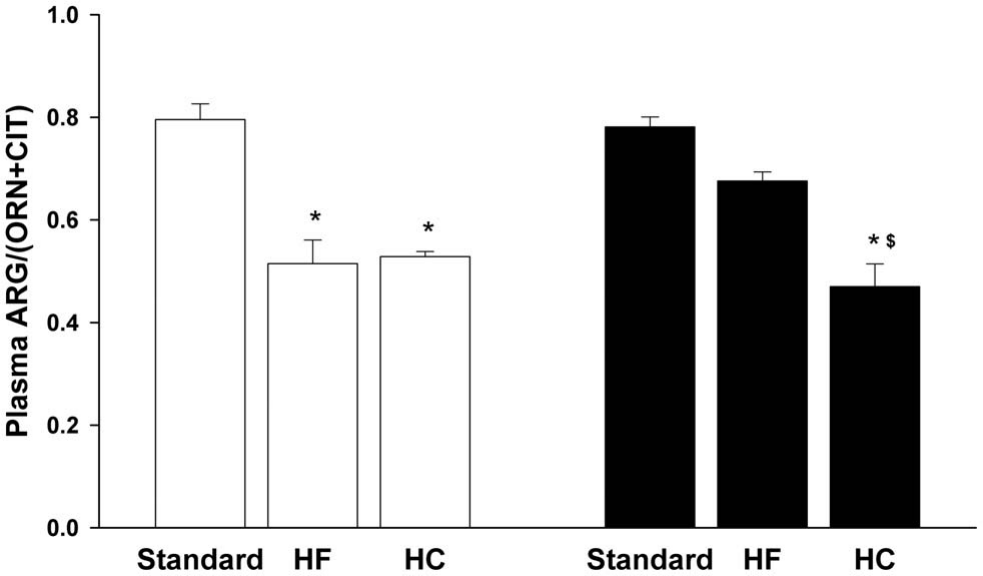
HF and HC diets reduce global arginine bioavailability. Values are means ± SE for n = 3–4 in each group. Abbreviations: ARG, arginine; ORN, ornithine; CIT, citrulline. Symbols are defined in Figure 2 legend.

Consideration of the plasma levels of these individual amino acids may fail to reveal significant changes in arginine metabolism because it overlooks the fact that they are metabolically interrelated [1]. Consequently, the ratio of plasma arginine to the sum of plasma ornithine plus citrulline has been developed as an indicator of global arginine bioavailability that is more sensitive to perturbations in arginine metabolism than are levels of the individual amino acids [23], [24]. Unlike the values of individual amino acids, this ratio clearly revealed effects of the diets: global arginine bioavailability was significantly reduced in C57BL6 fed the HC diet and apoE^−/−^ mice fed the HF or HC diet (Figure 5). The groups with reduced global arginine bioavailability also exhibited increases in plasma arginase activity and liver enzymes (Figures. 1 and 3).

### Effects of diet on tissue arginase activity and expression

**Figure 6:**
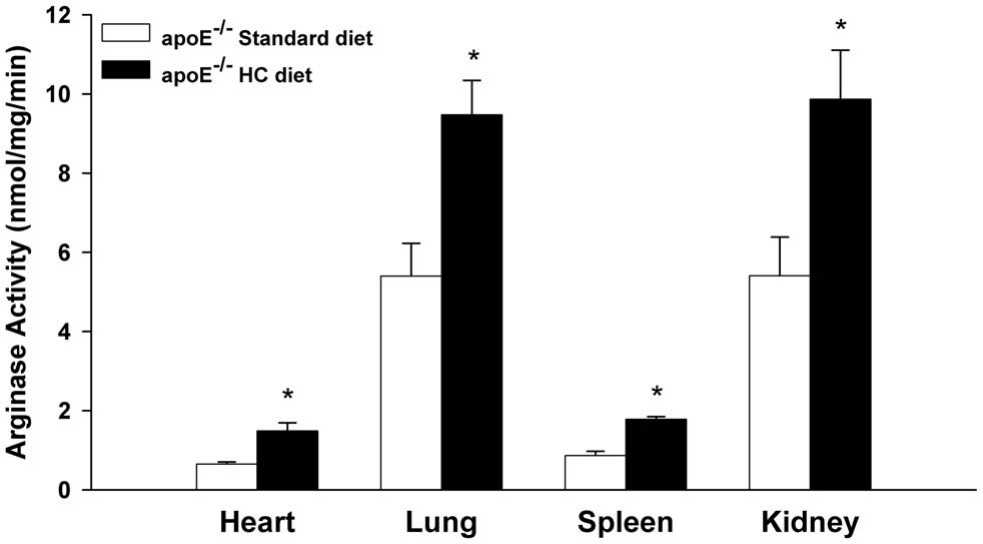
Induction of tissue arginase activities in apoE^−/−^ mice on HC diet. Values are means ± SE for n = 6 in each group. *p<0.05 vs standard diet.

In contrast to the changes in the plasma, arginase activities in tissues were increased only in the apoE^−/−^ mice fed the HC diet (Figure 6), the group with the highest plasma cholesterol levels. No increases were observed in apoE^−/−^ mice fed the HF diet or in any of the C57BL/6J groups (data not shown). Increased arginase activity was observed in the heart, lung, kidney and spleen of apoE^−/−^ mice fed the HC diet (Figure 6). The high levels of liver arginase activity did not differ significantly between strains and were not altered by any of the experimental conditions (e.g., apoE^−/−^ mice: 3584±507 nmol/mg/min; standard diet vs 3958±324 nmol/mg/min; HC diet), consistent with the fact that hepatic levels of arginase and the other urea cycle enzymes are responsive primarily to changes in the intake and catabolism of protein [31]–[33].

**Figure 7:**
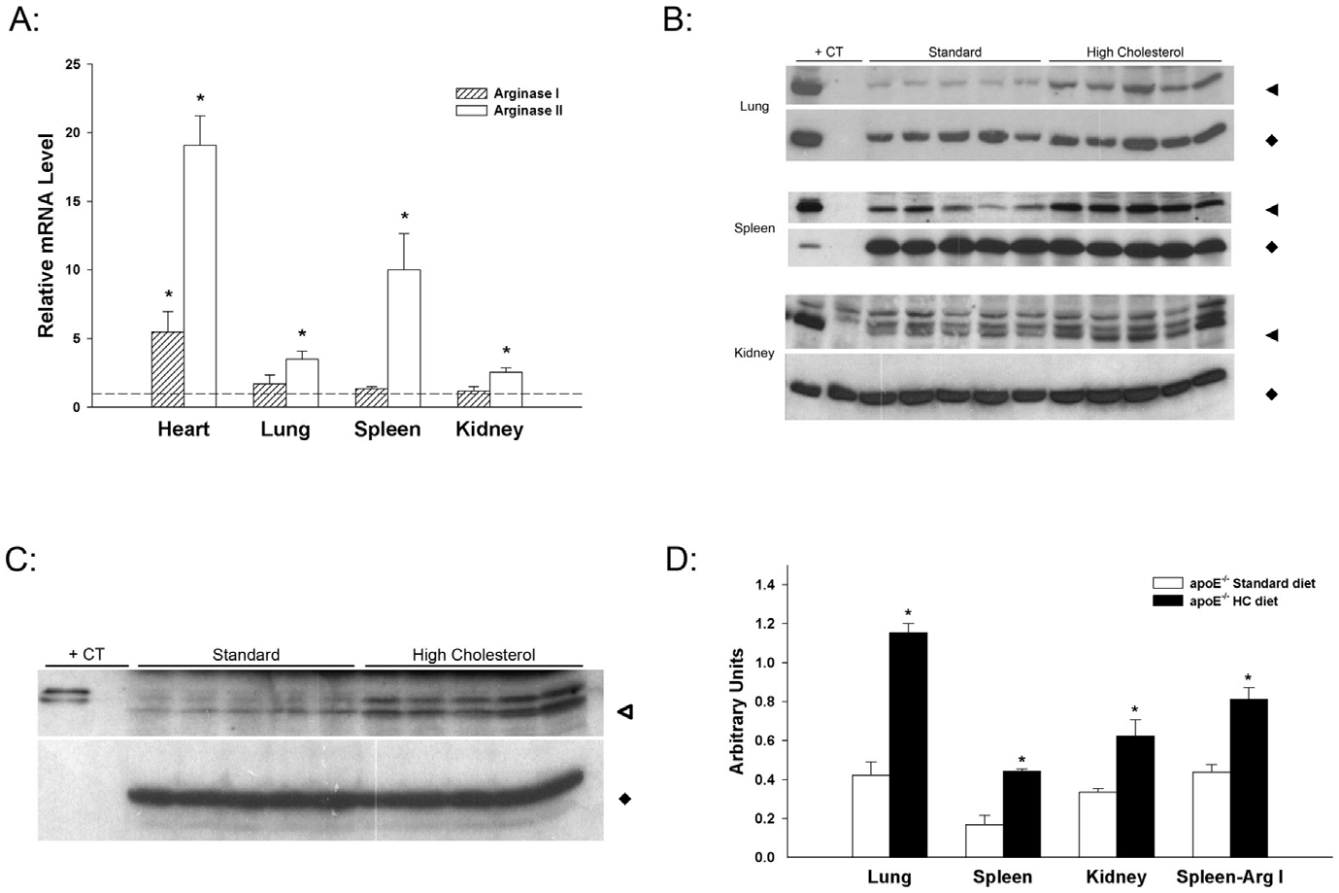
Induction of arginase mRNA and protein in apoE^−/−^ mice on HC diet. (A) Effect of HC diet on arginase I and II mRNAs in heart, lung, spleen and kidney. mRNA levels are expressed relative to the levels in apoE^−/−^ mice on standard diet (arbitrarily set to 1.0 for each tissue and indicated by dotted line). Values are means ± SE for n = 5–7 in each group. *p<0.05 vs standard diet. (B) Effect of HC diet on arginase II protein abundance in lung, spleen and kidney. For each tissue, the upper panel represents arginase II and the lower panel GAPDH. Western blots of extracts from tissues of 5 representative animals on each diet are shown. Amounts of protein loaded in each lane were 10 µg (lung and kidney) or 25 µg (spleen). An extract of C57BL/6J whole kidney (20 µg for lung and kidney blots, 3 µg for spleen blot) was used in the first lane of each blot as positive control (+CT) for arginase II. Twenty µg protein from kidney of the arginase II knockout mouse [47] was included in the second lane of the kidney blot in order to establish identity of the lowest band as arginase II. (C) Effect of HC diet on arginase I protein abundance in spleen. The upper panel represents arginase I and the lower panel GAPDH. The Western blot represents extracts from spleen (50 µg each lane) of 5 representative animals on each diet and 100 ng of C57BL/6J liver extract (+CT) as positive control. (D) Densitometry of Western blots, represented in arbitrary units (Arginase/GAPDH), was analyzed by Image Quant 5.2 Software. *p<0.01 vs standard diet. Molecular weights are indicated by the following symbols: solid triangles, 39 kDa; solid diamonds, 37 kDa; open triangles, 37 kDa.

As arginase activity represents the sum of contributions by arginases I and II, levels of arginase mRNAs and protein were evaluated. Increased arginase activity was accompanied by increased arginase II mRNA in heart, lung, spleen and kidney of apoE^−/−^ mice fed the HC diet (Figure 7A). Only the heart had significantly increased arginase I mRNA. Increased arginase activity and arginase II mRNA in lung, spleen, and kidney were accompanied by increased levels of arginase II protein (Figure 7B and D). Arginase I protein was increased in extracts from spleen (Figure 7C and D) but was below the level of detection in Western blots of lung and kidney extracts. Thus, arginase II was primarily responsible for the overall arginase activity in lung and kidney whereas both isoforms contributed to overall activity in spleen.

## Discussion

Although previous studies have implicated elevated arginase activity and expression in atherogenesis (reviewed in [2], [3], [34]), results of the present investigation demonstrate that an “atherogenic diet” can result in more widespread alterations in arginase activity and arginine metabolism than has previously been recognized. Major new findings of this study include demonstrations that the HC diet led to markedly elevated circulating arginase activity and reduced arginine bioavailability in both wild-type and apoE^−/−^ mice, and these changes occurred also in the apoE^−/−^ mice on the high fat diet. So far as we are aware, effects of high fat or high cholesterol diets on plasma arginase and global arginine bioavailability have not been evaluated in any previous studies. The elevation of plasma arginase activity correlated closely with increased plasma ALT levels, indicating that damage of the liver, which contains the greatest amount of arginase in the body, was primarily responsible for the elevated plasma arginase. If plasma arginase were derived significantly from tissues other than liver, it is probable that plasma values for ALT and arginase in apoE^−/−^ mice on the HC diet—the only condition in which arginase activity was significantly elevated in multiple tissues—would not correlate with plasma values for ALT and arginase in apoE^−/−^ mice on the HF diet. With the exception of a single outlier, however, the correlation was strong across all diet groups (Figure 4). Increased plasma arginase following liver injury has been well-documented by other investigators [35]–[38]. Although reductions in global arginine bioavailability in this study were consistently associated with elevations in plasma arginase activity rather than with increases in tissue arginase activity, this does not rule out the possibility that increased tissue arginase activity may additionally result in localized reductions in arginine availability that may impact vascular or tissue function.

Reductions in global arginine bioavailability are associated with cardiovascular complications and have been shown to be an independent risk factor for morbidity and mortality in humans [23], [24]. In sickle cell patients, reduced global arginine bioavailability is associated with pulmonary arterial hypertension [23], which leads to right ventricular hypertrophy that likely contributes to the increased mortality in this patient population. In non-sickle cell patients, reductions in global arginine bioavailability are associated with development of obstructive atherosclerotic coronary artery disease and increased incidence of major adverse cardiovascular events [24]. Taken together, these findings raise the possibility that diet-induced reductions in global arginine bioavailability alone may increase risk for developing some types of cardiovascular/endothelial dysfunction that may not be readily apparent except perhaps at longer time periods of dietary treatment, even in animal models that do not develop significant atherosclerotic plaques within a few months (e.g., mice fed HF diets).

As noted previously, mice fed the HC diet exhibit an accelerated rate of atherosclerotic development and hepatic inflammation that is correlated with increased plasma cholesterol. Interestingly, the HF diet did not raise plasma cholesterol levels but did result in liver injury in the apoE^−/−^ mice, as indicated by the elevated plasma arginase and ALT activities, but not in the C57BL/6J strain. In contrast to the HC diet, however, the HF diet did not lead to increased arginase activity in tissues of apoE^−/−^ mice.

Although both C57BL/6J and apoE^−/−^ mice maintained on the HC diet, which results in accelerated plaque development [21], [39], [40], exhibited increases in plasma arginase and reductions in global arginine bioavailability, the apoE^−/−^ mice fed the HC diet were the only animals with increases in tissue arginase activity and expression. Although C57BL/6J mice on the HC diet also had cholesterol levels that were significantly increased, previous studies have shown that the HC diet does not induce significant atheroma formation within the two-month time frame used in this study [21], [41]. It is therefore of particular interest that apoE^−/−^ mice fed the same percentage cholesterol as the HC diet used in our study had reduced plaque development when the apoE^−/−^ mutation was expressed in a mouse strain that completely lacks expression of arginase II [34]. The fact that multiple tissues in the apoE^−/−^ mice fed the HC diet had increased arginase activity and expression suggests that an atherogenic diet, via increased arginase expression, may have a broader impact on vascular and organ function than has previously been appreciated.

The fact that tissue arginase was elevated only in apoE^−/−^ mice on the HC diet raises the possibility that the mechanism(s) underlying increased arginase expression also may be part of the inflammatory process leading to atherosclerosis. It is known that arginase is increased in response to a variety of inflammatory stimuli [3], [7]–[9], but the precise stimuli involved in arginase induction under atherogenic conditions have not been identified. Since activation of liver X receptors by cholesterol metabolites has been shown to induce expression of the arginase II gene [42], it is possible that cholesterol metabolites may themselves play a direct role in inducing arginase II expression in tissues of apoE^−/−^ mice fed the HC diet.

In summary, these findings demonstrate for the first time that increased plasma arginase activity and widespread tissue expression of arginase II–together with reductions in global arginine bioavailability–are associated with a diet that induces atherogenesis in apoE^−/−^ mice. Further studies are warranted to elucidate the possible roles of increased circulating arginase, reduced global arginine bioavailability, and elevated tissue arginase activity in vascular dysfunction, as well as to identify the specific cells in tissues in which arginase expression was increased and to elucidate their impact on vascular function.

## Methods

### Experimental Design

Male C57BL/6J and B6.129P2-Apoe^tm1Unc^ (apoE^−/−^ mice) (Jackson Laboratory, Bar Harbor, ME) aged 6–7 weeks were used in this study. All mice were provided water *ad libitum* in ventilated cages in a controlled humidity and temperature environment with a 12hr light/dark cycle. Animal care and use procedures were conducted in accordance with the “PHS Policy on Human Care and Use of Laboratory Animals” and the “Guide for the Care and Use of Laboratory Animals” (NIH publication 86–23, 1996). These procedures were approved by the National Institute for Occupational Safety and Health Institutional Animal Care and Use Committee (internal approved protocol ID 07-PS-M-014). In three independent experiments, different groups of mice were placed on one of the following diets for 2 months: 1) standard chow diet (Harlan 7913 irradiated NIH-31 6% rodent diet containing protein, 186 g/Kg; fat, 62.5 g/Kg; fiber, 45.3 g/Kg; metabolizable energy, 3.13 Kcal/g; 18% total kcal from fat; 0.053% cholesterol), 2) high fat and high cholesterol diet containing cholate (HC; TD88051 Harlan; 37.1% total kcal from fat; 1.25% cholesterol; 0.5% sodium cholate) [43] and 3) high fat diet (HF; D12451 Research Diets; 45% total kcal from fat; 0.20% cholesterol) [44]. Prior to sacrifice and collection of blood mice were fasted 16 hr. Following CO_2_ asphyxiation blood was collected from vena cava followed by perfusion with cold PBS via the heart. For one group of experimental animals in which responses to all 3 diets were evaluated, tissues also were harvested, quick frozen in liquid nitrogen and stored at −80°C for arginase activity and RNA isolation.

### Plasma Chemistry

In initial experiments we found that plasma from some samples of whole blood that had been placed on ice for a couple of hours prior to fractionation had remarkably low arginine (<20 µM) and high ornithine levels (>150 µM) that were not representative of values in plasma that was rapidly fractionated and frozen. Thus, EDTA blood samples were *immediately* centrifuged, and plasma was quick-frozen and stored at −80°C until used for analysis. Plasma levels of total cholesterol, triglycerides, blood urea nitrogen and ALT were analyzed (AniLytics Inc.). A complete plasma amino acid profile was determined by the Medical Genetics Laboratory at Baylor College of Medicine.

### Arginase Activity

For arginase activity, tissue homogenates were prepared using the TissueLyser (Qiagen) in 0.1% Triton X-100 with 2 mM Pefabloc (Roche), 2 µg/ml pepstatin A (Sigma) and 10 µg/ml leupeptin (Sigma). Samples were placed on ice for 10 minutes then centrifuged at 12,000 g for 10 minutes and supernatants were used. Arginase activity in plasma and tissue homogenates was determined as the conversion of [^14^C-guanidino]-L-arginine (American Radiolabeled Chemicals or Moravek) to [^14^C]urea (2.5-hr incubation for plasma and all tissues except liver – 1/50 dilution for 30 min), which was then converted to ^14^CO_2_ by urease and trapped as Na_2_^14^CO_3_ and counted on a scintillation counter as described previously [8]. Protein concentrations were determined by the BCA protein assay kit (Pierce) and used to calculate arginase specific activities. Plasma arginase activity was expressed as activity per volume plasma (nmol/ml/min).

### Real-time RT-PCR

Real-time RT-PCR was performed as previously described [45]. Briefly, RNA was isolated using the RNeasy Mini Kit (Qiagen) according to manufacturer’s directions. One µg of total RNA was reverse transcribed using random hexamers (Applied Biosystems) and Superscript III (Invitrogen). Five µl of cDNA (in duplicate for each gene) was then used for mRNA determinations. Pre-designed Assays-on-Demand™ TaqMan^®^ gene expression assays (Applied Biosystems) for arginase I and arginase II were used. Ribosomal 18S RNA and glyceraldehyde 3-phosphate dehydrogenase (GAPDH) mRNA were used as internal references for normalization. Relative gene expression was calculated using the comparative threshold method (2^−ΔΔCt^) [46]. There was no difference in relative fold change compared to reference using either 18S rRNA or GAPDH mRNA.

### Western Blot

Using methods described previously [45], Western blotting was performed on the tissue homogenates used for assays of arginase activity. Western blots were developed with antibody to arginase I (BD Transduction Laboratories #610708;1∶1000 dilution in 1% nonfat milk/1%BSA/Tris-buffered saline containing 0.1% Tween 20 (TBS-T)), antibody to arginase II (Santa Cruz Biotechnology #sc-20151; 1∶500 dilution in 5% nonfat milk in TBS-T) and secondary HRP-linked antibodies (1∶5000) from GE Healthcare. Following immunodetection of arginase, blots were stripped (Pierce Restore Plus Western Blot Stripping Buffer) and re-probed with antibody to GAPDH (Santa Cruz Biotechnology #sc-25778; 1∶2000 dilution in 5% milk/TBS-T) to verify equal protein loading. There were insufficient amounts of heart extract to analyze by Western blot.

### Statistics

All data are presented as mean ± standard error. Analyses were performed using JMP^®^ Statistical Discovery Software utilizing analysis of variance with a least squares means table generated by a student’s t-test post-hoc test. In data sets where there was no homogeneity of variance, values were log transformed prior to analysis. Student’s t-test was used only when two groups of equal sample size were compared. Differences were considered statistically significant at p<0.05.
